# Health literacy and its correlates among adults surveyed in Kuwait: a venue-based cross-sectional study

**DOI:** 10.3389/fpubh.2026.1878619

**Published:** 2026-07-08

**Authors:** Ahmad Salman, Fatima Al-Ghadban, Wafaa Al-Kandari, Nadia Jumah

**Affiliations:** 1 Department of Public Health Practice, College of Public Health, Kuwait University, Kuwait City, Kuwait; 2 Non-Communicable Disease Prevention and Control Directorate, Ministry of Health, Kuwait City, Kuwait; 3 School Health Directorate, Ministry of Health, Kuwait City, Kuwait; 4 Assistant Undersecretary for Health Care Affairs, Ministry of Health, Kuwait City, Kuwait

**Keywords:** community health education, health communication, health equity, health literacy, health literacy responsiveness, Kuwait, mental health literacy, non-communicable diseases

## Abstract

**Background:**

Health literacy is a foundational determinant of health equity, yet remains understudied in Kuwait and the broader Gulf region. This venue-based survey assessed health literacy and its sociodemographic and health-related correlates among adults surveyed in Kuwait, to inform equity-oriented health communication and community health education.

**Methods:**

A venue-linked, electronic cross-sectional survey was conducted across all six governorates of Kuwait (February–April 2026). Adults aged 18 years or older were recruited using quota-informed convenience sampling through an electronic self-administered questionnaire distributed at selected public venues and study channels. Validated Arabic and English versions of the HLS-EU-Q16 were administered, yielding 1,846 participants (81.1% Kuwaiti; 78.4% female). Associations were examined using ANOVA (including Welch), Kruskal–Wallis tests, general linear modelling, and logistic regression, with sensitivity analyses for the sparse poor-SRH category.

**Results:**

The mean HLS-EU-Q16 score was 12.89 (SD = 3.42; Cronbach’s *α* = 0.849), and 37.2% of participants surveyed had limited health literacy (inadequate 10.6%; problematic 26.5%). Finding information on mental illness was the hardest task (C8: 57% rated it easy). Self-rated health (SRH) was the factor most strongly associated with limited health literacy, showing a graded gradient robust to tests not assuming equal variances or normality (Welch and Kruskal–Wallis both *p* < 0.001). The association persisted in the adjusted logistic model; because only 12 participants reported poor SRH, a model collapsing poor and fair SRH gave a stable adjusted odds ratio of 4.91 (95% CI 3.00–8.03). Increasing age was independently associated with lower odds (aOR = 0.989/year, *p* = 0.010), whereas a sociodemographic-only model was non-significant (Nagelkerke *R*^2^ = 0.011, *p* = 0.217).

**Conclusion:**

Over one-third of the adults surveyed in Kuwait had limited health literacy. Self-rated health was the strongest correlate in the adjusted model, though the cross-sectional design precludes causal inference. Mental health information access represents a specific, actionable gap. These findings call for accessible, culturally and linguistically appropriate community health education, system-level responsiveness, and communication strategies prioritising individuals with poorer perceived health and chronic conditions. Because the sample was venue-recruited and predominantly Kuwaiti and female, estimates apply to the surveyed population rather than all residents of Kuwait.

## 1 Introduction

Health literacy is a well-established determinant of health and a cornerstone of effective public health practice. It is defined as the knowledge, motivation, and competencies required to access, understand, appraise, and apply health information in order to make judgments and decisions in everyday life concerning healthcare, disease prevention, and health promotion (1). Beyond knowledge and skills, contemporary definitions emphasise the capacity to navigate increasingly complex health systems and digital information environments (2, 3). This broader conceptualisation reflects the growing recognition that health literacy is not merely an individual attribute but a contextually determined capability shaped by social, cultural, and structural factors (2).

The significance of health literacy lies in its strong and well-documented associations with health behaviours, service utilisation, and health outcomes (4). Limited health literacy contributes to inequitable health outcomes by compounding the effects of socioeconomic disadvantage and reduced access to quality health information (4–6). Individuals with limited health literacy are more likely to misuse medications, attend emergency services unnecessarily, manage chronic conditions suboptimally, and experience higher rates of preventable hospitalisation (4, 5). These consequences are particularly salient in populations managing non-communicable diseases (NCDs), where self-management demands are high and information needs are complex (7). Strengthening health literacy is therefore a cornerstone of community empowerment and community-centred health education.

In addition to traditional health literacy demands, the modern health information environment has introduced new challenges. As individuals are exposed to growing volumes of health information through digital channels, the ability to critically appraise and apply such information—often termed eHealth literacy—has become increasingly relevant (8). This is especially important in settings such as Kuwait, where smartphone penetration and social media use are among the highest globally, yet the quality of health information circulating through these channels is inconsistent.

In the Middle East, health literacy has received growing attention, yet remains insufficiently assessed in many countries, including Kuwait (9). Non-communicable diseases—particularly type 2 diabetes, hypertension, and obesity—represent a pressing public health challenge across the Gulf Cooperation Council (GCC) region (10). Kuwait has among the highest rates of adult obesity globally (11) and carries a substantial and growing cardiometabolic burden, with diabetes and hypertension remaining leading causes of premature mortality (10, 12, 13). Health literacy is a direct determinant of the population’s capacity to prevent and manage these conditions effectively (14).

Kuwait presents a particularly important context for this investigation. The country carries one of the highest NCD burdens in the region, and despite ongoing health system expansion, health promotion infrastructure and research capacity remain areas of active development (14, 15). Its population is uniquely diverse: as of December 2025, approximately 31.4% of residents are Kuwaiti nationals and 68.6% are expatriates, predominantly from South and Southeast Asia (16). This demographic structure creates a health literacy environment of considerable heterogeneity, with potential disparities in language access, health system navigation, and cultural health beliefs across subpopulations that have not been systematically characterised.

Existing evidence on health literacy in Kuwait is limited in scope and predominantly restricted to clinical populations. Hussein et al. (17) reported that 44.5% of Kuwaiti adults with type 2 diabetes had inadequate health literacy using a locally adapted tool; a subsequent study in the same cohort found that limited health literacy was independently associated with poor glycaemic control (18). No large-scale venue-linked Arabic/English assessment using an internationally validated instrument has been conducted in Kuwait, and the sociodemographic and health-related correlate profile of limited health literacy in broader adult survey samples remains insufficiently characterised.

The European Health Literacy Survey Questionnaire (HLS-EU-Q) and its validated 16-item short form, the HLS-EU-Q16, provide a robust and widely used framework for measuring health literacy across three domains: healthcare, disease prevention, and health promotion (19). The instrument has been validated across multiple European and Asian settings (20) and the Arabic version has demonstrated good psychometric properties in Arabic-speaking populations (21). Its use in Kuwait enables direct cross-cultural and cross-regional comparisons, supporting the generation of internationally relevant evidence.

Given Kuwait’s high NCD burden, diverse resident population, and absence of community-level health literacy data, a large-scale assessment is needed to inform accessible, equity-oriented community health education. This study aims to: (1) estimate the proportion of surveyed adults with limited health literacy among Arabic- and English-literate adults surveyed at public venues across Kuwait using the HLS-EU-Q16; (2) characterise item-level difficulty across the 16 tasks, with particular attention to areas relevant for community health communication; and (3) identify the sociodemographic, health-related, and behavioural factors associated with limited health literacy in an adjusted multivariable model, to support priority-setting for community-centred health education and system-level responsiveness.

## 2 Materials and methods

### 2.1 Study design

This study employed a cross-sectional survey design to assess health literacy among adults surveyed in Kuwait. Data were collected using a structured electronic self-administered questionnaire distributed at selected public venues and approved electronic distribution channels (22, 23). The study is reported in accordance with the STROBE statement for observational studies (24). Although quotas were used to improve demographic coverage, the sample should not be interpreted as nationally representative in the strict probabilistic sense.

### 2.2 Study setting and period

Data collection was conducted across all six governorates of Kuwait—Al Asimah (Capital), Hawalli, Al Farwaniyah, Al Ahmadi, Al Jahra, and Mubarak Al-Kabeer—between February and April 2026. The electronic questionnaire was distributed through selected public venues and approved electronic distribution channels. Distribution points and channels included government-operated community centres, primary healthcare clinics, university campuses, shopping centres, and relevant community networks, selected to encourage demographic diversity and reach both Kuwaiti nationals and expatriate communities.

### 2.3 Study population and eligibility

The target population comprised adults aged 18 years or older residing in Kuwait at the time of data collection, regardless of nationality, who were able to complete the questionnaire in Arabic or English. Of 1,944 questionnaire records assessed, 98 were excluded during data cleaning and validation (incomplete consent, age below 18 years, or fewer than 14 of 16 HLS-EU-Q16 items answered), yielding a final analytic sample of 1,846 participants. Internet-use frequency data were missing for 79 participants (4.3%); regression models including this covariate were therefore conducted among the 1,767 participants with complete data. A participant flow diagram is provided in Supplementary Figure S1.

### 2.4 Sample size

The minimum required sample size was calculated using the standard formula for estimating a proportion [*n* = *Z*^2^*p*(1 − *p*)/*d*^2^], assuming a 95% confidence level (*Z* = 1.96) and a 5% margin of error (*d* = 0.05). Hussein et al. (17) is, to the authors’ knowledge, the only available published study reporting a health literacy prevalence estimate in Kuwait. As no general-population estimate exists, this clinical prevalence of 44.5% among Kuwaiti adults with type 2 diabetes was used as the most conservative locally derived figure. Applying this proportion with an additional 10% allowance for non-response yielded a minimum sample of 423 participants. A target of 500–600 was set to ensure adequate statistical power for subgroup analyses (19). The final achieved sample of 1,846 substantially exceeded this minimum, providing robust power for all planned analyses.

### 2.5 Sampling strategy

A quota-informed convenience sampling strategy was used to improve geographic and demographic coverage while maintaining practical feasibility (22). Quota targets were set by governorate, nationality, and age group to encourage diversity of recruitment. Recruitment was conducted through distribution of the electronic questionnaire link across selected public venues and approved electronic distribution channels, with monitoring of responses across governorate, nationality, and age-group strata where feasible. In practice, quota targets were not uniformly achieved: the final sample over-represented Kuwaiti nationals (81.1%) and women (78.4%) relative to the resident population, in which Kuwaiti nationals constitute approximately 31% (16). Detailed numeric recruitment targets and refusal counts were not systematically retained; because the survey link was distributed electronically, the number of individuals who viewed but did not complete the questionnaire could not be determined, and a formal response rate cannot be calculated. The sample is therefore best understood as a large venue-linked, electronic, Arabic- and English-language survey sample rather than a nationally representative probability sample.

### 2.6 Measurement instrument

Health literacy was measured using the validated 16-item European Health Literacy Survey Questionnaire (HLS-EU-Q16). The instrument assesses self-reported ease or difficulty across three core health literacy domains: healthcare, disease prevention, and health promotion. Each item uses a four-point response scale (very difficult, difficult, easy, very easy), dichotomised for scoring such that difficult and very difficult are coded 0, and easy and very easy are coded 1 (25). The sum score ranges from 0 to 16, with higher scores indicating greater health literacy, and participants are classified as inadequate (0–8), problematic (9–12), or sufficient (13–16) (25). The validated Arabic version of the HLS-EU-Q16 was used for participants completing the questionnaire in Arabic; this version has demonstrated acceptable reliability and validity in Arabic-speaking populations (21), consistent with psychometric evaluations of the HLS-EU-Q16 among other Arabic-speaking groups (26). The English-language HLS-EU instrument has been validated across multiple international settings, including Asian countries (20). In the present sample, internal consistency was high (Cronbach’s *α* = 0.849).

### 2.7 Additional variables

Sociodemographic variables collected included: age (continuous, in years), gender (male/female), nationality (Kuwaiti/Non-Kuwaiti/Bedoon), governorate of residence, marital status (single/married/divorced/widowed), highest educational attainment (six levels, recoded to four for regression), and employment status. Monthly household income was recorded in five Kuwaiti Dinar (KD) bands. Health-related variables included: self-reported chronic disease (any/none, and type), self-rated health (SRH; five-point scale from poor to excellent), and frequency of internet use for health information (five-point scale, recoded to three levels). Trust in health information sources was also recorded (27). The referent physician or healthcare professional was identified as the most trusted source by the majority.

### 2.8 Data collection procedure

Data were collected using a structured electronic self-administered questionnaire. The questionnaire was available in Arabic and English, and participants self-selected their preferred language version. Informed consent was obtained electronically before questionnaire completion. In the final analytic sample, the majority completed the Arabic version (*n* = 1,655; 89.7%), while 191 participants (10.3%) completed the English version. Responses from both language versions were merged into a single dataset, and all age entries were cleaned programmatically to resolve Arabic numeral formats, written word forms, and birth-year entries.

### 2.9 Data analysis

Statistical analyses were performed using IBM SPSS Statistics (version 31). Descriptive statistics were computed for all variables. The Cronbach’s *α* coefficient was calculated to assess internal consistency of the HLS-EU-Q16. HLS-EU-Q16 sum scores were calculated for participants with at least 14 of 16 items answered, and used to classify individuals into the three HL categories. Health literacy scores are reported as means with standard deviations.

Bivariate associations between HLS score (continuous) and predictor variables were assessed using independent-samples *t*-tests (binary predictors) and one-way ANOVA with *post-hoc* Bonferroni correction (categorical predictors). Spearman’s rank correlation was computed for ordinal variables. Assumptions underlying the ANOVA and general linear model analyses were assessed using Levene’s test of equality of error variances and inspection of residual and normal probability plots. Because these checks indicated heterogeneity of variance and non-normal residuals for the bounded HLS-EU-Q16 score, key bivariate comparisons were re-examined using Welch’s ANOVA with Games–Howell *post-hoc* tests and the Kruskal–Wallis test (with the Mann–Whitney *U* test for binary comparisons) as distribution-free and variance-robust sensitivity analyses; given the large sample size, the parametric *F*-tests were also expected to be reasonably robust. Binary logistic regression was performed to identify factors independently associated with limited health literacy (the combined inadequate/problematic category), reporting: (a) crude odds ratios for each predictor; (b) a fully adjusted multivariable model; and (c) a hierarchical block-entry model to assess incremental variance explained. Logistic model fit was assessed using the Hosmer–Lemeshow test and Nagelkerke *R*^2^ (28). Because only 12 participants reported poor self-rated health, a sensitivity analysis collapsing the poor and fair SRH categories was conducted to address potential sparse-data bias in this category. A general linear model (GLM) with Type III sums of squares was used to estimate adjusted effects on the continuous HLS score (29). For regression analyses, education was collected at six levels and recoded to four (below secondary, secondary/diploma, bachelor’s, postgraduate), and internet use was recoded from five to three levels (low, moderate, high), to ensure adequate cell sizes. Internet-use frequency was missing for 79 participants (4.3%); models including this covariate were conducted among 1,767 participants with complete data (complete-case analysis). Income data were complete for all participants. Analyses were performed in IBM SPSS Statistics version 31, with statistical significance set at *p* < 0.05.

### 2.10 Ethical considerations

Ethical approval was granted by the Standing Committee for Coordination of Health and Medical Research at the Kuwait Ministry of Health on 17 February 2026 (Research No.: 3079/2025; Ministry Ref: 2026/84). All participation was voluntary and anonymous. No personally identifiable data were collected. Electronic informed consent was obtained from all participants prior to questionnaire administration.

## 3 Results

### 3.1 Sample characteristics

A total of 1,846 adults were included in the final analysis (Table 1). The mean age was 37.58 years (SD = 14.53; range 18–96). Participants were predominantly female (78.4%), Kuwaiti nationals (81.1%), educated to at least bachelor’s degree level (59.2%), and married (54.6%). More than seven in ten reported a monthly household income of 1,000 KD or above. A quarter (24.5%) had at least one chronic disease, most commonly hypertension (10.8%) and diabetes (9.4%). Most rated their health favourably—72.5% described it as very good or excellent—while only 5.2% rated it as fair or poor.

**Table 1:** Sociodemographic and health characteristics of the study sample (*n* = 1,846).

Variable	Category	*n*	%	Cum. %
Age (years)	Mean ± SD = 37.58 ± 14.53; range 18–96			
Gender	Female	1,447	78.4	
	Male	399	21.6	
Nationality	Kuwaiti	1,497	81.1	
	Non-Kuwaiti (expatriate)	314	17.0	
	Bedoon (stateless)	35	1.9	
Governorate	Al Asimah (capital)	466	25.2	
	Hawalli	349	18.9	
	Al Farwaniyah	296	16.0	
	Al Ahmadi	252	13.7	
	Al Jahra	249	13.5	
	Mubarak Al-Kabeer	234	12.7	
Marital status	Married	1,008	54.6	
	Single	670	36.3	
	Divorced	125	6.8	
	Widowed	43	2.3	
Education	Bachelor’s degree	965	52.3	
	Secondary/diploma	710	38.5	
	Postgraduate	127	6.9	
	Below secondary (combined)	44	2.4	
Employment	Employed	848	45.9	
	Student	406	22.0	
	Retired	350	19.0	
	Homemaker	120	6.5	
	Unemployed	80	4.3	
	Self-employed	42	2.3	
Income (KD/month)	2,000 + KD	524	28.4	
	1,000–1,499 KD	502	27.2	
	1,500–1,999 KD	299	16.2	
	<500 KD	278	15.1	
	500–999 KD	243	13.2	
Chronic disease	None	1,393	75.5	
	Any chronic disease	453	24.5	
	Hypertension	200	10.8	
	Diabetes	174	9.4	
	Obesity	123	6.7	
	Heart disease	96	5.2	
	Asthma/lung disease	65	3.5	
Self-rated health	Very good	825	44.7	
	Excellent	514	27.8	72.5
	Good	412	22.3	
	Fair	83	4.5	5.2
	Poor	12	0.7	
Internet use for health	Moderate (sometimes)	755	42.7	
	High (often/daily)	729	41.3	
	Low (never/rarely)	283	16.0	

KD, Kuwaiti Dinar. Internet-use frequency was missing for 79 participants (4.3%); internet-use percentages are calculated from valid responses (*n* = 1,767). Income data were complete for all 1,846 participants. Participants could report more than one chronic disease.

### 3.2 Health literacy levels

The HLS-EU-Q16 demonstrated strong internal consistency (Cronbach’s *α* = 0.849). The mean total score was 12.89 (SD = 3.42; median = 14.0; range 0–16). Nearly two-thirds of participants (62.8%, *n* = 1,160) achieved sufficient health literacy. However, 37.2% of those surveyed — over one in three — had limited health literacy (inadequate: 10.6%; problematic: 26.5%), highlighting a substantial public health concern within this venue-based sample. Full score distribution is presented in Table 2.

**Table 2:** HLS-EU-Q16 score distribution and item-level analysis (*n* = 1,846).

HLS-EU-Q16 Item/Category	Description	*n* (%) or % easy
Score distribution		
Sufficient (13–16)		1,160 (62.8%)
Problematic (9–12)		490 (26.5%)
Inadequate (0–8)		196 (10.6%)
Limited HL total (0–12)		**686 (37.2%)**
Mean score (SD); median		12.89 (3.42); 14.0
Cronbach’s α		0.849
Item difficulty—% rating task easy or very easy	Ranked easiest to hardest	
C4	Understanding medication instructions	93%
C3	Understanding what the doctor says	91%
C7	Following doctor/pharmacist instructions	90%
C9	Understanding health warnings (e.g., smoking)	86%
C1	Finding information on treatments for illness	85%
C13	Finding info on mental wellbeing activities	84%
C15	Understanding health information in the media	83%
C14	Understanding advice from family or friends	81%
C2	Finding professional help when ill	80%
C6	Using the doctor’s information to make decisions	79%
C12	Finding information on illness prevention	79%
C16	Judging which lifestyle habits affect health	79%
C10	Understanding why screening tests are needed	78%
C5	Deciding whether to seek a second opinion	72%
C11	Judging if media health information is reliable	70%
*C8*	*Finding information on mental illness (depression, stress)*	*57%—13 pp gap*

HL, health literacy. Limited HL = inadequate (0–8) + problematic (9–12). C8 was the hardest item by 13 percentage points (13 pp below C11: 70%), representing the largest inter-item gap in the scale. Items ranked from easiest (C4) to hardest (C8). Bold type denotes the overall prevalence of limited health literacy (the combined inadequate and problematic categories).

Item-level analysis showed that most clinical tasks were well-managed, but mental health information access was a clear and specific exception. While understanding medication instructions (C4: 93%) and understanding the doctor (C3: 91%) were rated easy by the large majority, only 57% found it easy to find information on how to deal with mental illness such as depression or stress (C8)—the hardest item by a margin of 13 percentage points over the next hardest task (judging media reliability, C11: 70%). This gap was consistent across subgroups.

### 3.3 Bivariate associations with health literacy

Self-rated health was the factor most strongly associated with health literacy scores, demonstrating a clear and graded association (*F* = 22.388, *p* < 0.001; Spearman *ρ* = 0.219, *p* < 0.001). Mean HLS scores rose monotonically from 8.50 among those rating their health as poor to 13.70 among those rating it as excellent—a 5.20-point range spanning 33% of the scale. Because Levene’s test indicated unequal variances, the association was re-examined using tests that do not assume variance homogeneity or normality; the graded gradient remained highly significant under Welch’s ANOVA (*F* = 17.438, df1 = 4, df2 = 76.425, *p* < 0.001) and the Kruskal–Wallis test (*H* = 94.754, df = 4, *p* < 0.001). Games–Howell *post-hoc* comparisons confirmed a graded pattern, with significant differences across the better-populated categories; comparisons involving the small poor-health group (*n* = 12) were less precise. No other variable approached this magnitude of association (full sensitivity results in Supplementary Table S1).

Four further variables were significantly linked to health literacy scores. Marital status (*F* = 4.738, *p* = 0.003) revealed a counterintuitive pattern: divorced participants recorded the highest mean score in the sample (13.83), significantly exceeding both single (*p* = 0.002) and married (*p* = 0.044) participants. Employment was a further significant factor (*F* = 3.318, *p* = 0.005), driven by students scoring lower than employed adults (12.41 vs. 13.19; *p* = 0.002). The youngest age group (18–29 years; mean 12.61) scored lower than those aged 30–44 years (13.25; *p* = 0.007)—a finding that was weaker and less consistent in robustness analyses (see Results sensitivity analyses and Supplementary Table S1). Chronic disease was linked to lower scores (12.52 vs. 13.02; *t* = 2.711, *p* = 0.007). By contrast, gender, nationality, education level, income, internet use, and governorate were all non-significant (all *p* > 0.145). Full bivariate results are presented in Table 3.

**Table 3:** Mean HLS-EU-Q16 score by participant characteristics—bivariate analysis (*n* = 1,846).

Variable	*n*	Mean (SD)	Statistic	*p*	*Post-hoc* (Bonferroni)
Gender					
Male	399	12.78 (3.55)	*t = −0.742*	0.458	NS
Female (ref)	1,447	12.93 (3.38)			
Nationality					
Kuwaiti (ref)	1,497	12.95 (3.33)	*F = 1.357*	0.258	All NS
Non-Kuwaiti	314	12.61 (3.81)			
Bedoon	35	13.00 (3.46)			
Age group					
18–29 years	652	12.61 (3.69)	*F = 3.623*	0.013	18–29 < 30–44 (*p* = 0.007)
30–44 years (highest)	571	**13.25 (3.07)**			
45–59 years	469	12.89 (3.36)			Other pairs NS
60+ years	154	12.79 (3.57)			
Education					
Below secondary	44	11.98 (4.02)	*F = 1.320*	0.253	All NS
Secondary/diploma	710	12.72 (3.56)			
Bachelor’s (ref)	965	13.04 (3.21)			
Postgraduate	127	13.03 (3.40)			
Employment					
Employed (ref)	848	**13.19 (3.26)**	*F = 3.318*	0.005	Employed > student (*p* = 0.002)
Student	406	12.41 (3.54)			Other pairs NS
Retired	350	12.85 (3.45)			
Homemaker	120	12.56 (3.55)			
Unemployed	80	13.11 (3.82)			
Marital status					
Divorced (highest)	125	**13.83 (2.98)**	*F = 4.738*	0.003	Divorced > single (*p* = 0.002)
Married (ref)	1,008	12.96 (3.30)			Divorced > married (*p* = 0.044)
Single	670	12.64 (3.60)			
Widowed	43	12.44 (3.98)			
Income (KD/month)					
<500 KD	278	12.47 (3.81)	*F = 1.710*	0.145	All NS
2,000+ KD (ref)	524	13.13 (3.39)			
Chronic disease					
No (ref)	1,393	13.02 (3.35)	*t = 2.711*	0.007	Lower scores in chronic disease group
Yes	453	12.52 (3.59)			
Self-rated health (ref: excellent)					
Excellent (ref)	514	**13.70 (3.26)**	*F = 22.388*	<0.001	Graded SRH pattern; see Supplementary Table S1.
Very good	825	12.97 (3.14)			
Good	412	12.20 (3.68)			5.20-point range (poor → excellent)
Fair	83	11.20 (3.71)			
Poor (lowest)	12	**8.50 (5.04)**			
Internet use for health					
High use (often/daily)	729	12.88 (3.53)	*F = 0.883*	0.414	All NS
Moderate (ref)	755	12.87 (3.28)			
Low use (never/rarely)	283	13.16 (3.20)			

Self-rated health was the dominant bivariate predictor. All *post-hoc* tests are Bonferroni-corrected. NS, not significant. Ref, reference category. KD, Kuwaiti Dinar.

For self-rated health, the table reports Bonferroni-corrected *post-hoc* comparisons; robust Games–Howell results are provided in Supplementary Table S1, and comparisons involving the small poor-SRH group (*n* = 12) were less precise. Bold type identifies the highest or lowest mean HLS-EU-Q16 score within variables with a statistically significant bivariate association. Italic type is used for statistical notation and Bonferroni-adjusted pairwise *p*-values.

### 3.4 Adjusted analyses

#### 3.4.1 General linear model

The multivariable GLM (*n* = 1,767) was statistically significant and explained a modest proportion of variance in HLS scores (*F* = 5.904, *p* < 0.001, *R*^2^ = 0.057). Self-rated health was the factor most strongly associated with the HLS score, demonstrating a clear graded association after full covariate adjustment (*F* = 18.618, *p* < 0.001, partial *η*^2^ = 0.041) and the largest effect size among the variables examined. Age was the only other significant factor: each additional year was associated with a 0.020-point higher HLS score (95% CI 0.008–0.033; *F* = 10.264, *p* = 0.001, partial *η*^2^ = 0.006). Chronic disease approached but did not reach significance (*F* = 2.962, *p* = 0.085). Gender, nationality, education, income, and internet use were all non-significant after adjustment (all *p* > 0.263). Full model results are presented in Table 3.

A hierarchical sensitivity regression confirmed this pattern: sociodemographic variables alone explained less than 1% of variance (*R*^2^ = 0.007). Adding SRH and other health-related variables increased explained variance sevenfold (Δ*R*^2^ = 0.046; total *R*^2^ = 0.052, *p* < 0.001). Variance inflation factors were below 1.5 for all predictors, ruling out multicollinearity.

#### 3.4.2 Binary logistic regression

Table 4 presents crude and adjusted odds ratios for limited health literacy. In unadjusted analyses, self-rated health exhibited the most striking association, with a steep and monotonic graded gradient across all five levels. Participants rating their health as poor had nearly 15 times the unadjusted odds of limited HL compared with those rating it as excellent (crude OR = 14.618, 95% CI 3.162–67.583, *p* < 0.001). Students had 51% higher crude odds than employed adults (crude OR = 1.512, 95% CI 1.186–1.927) and homemakers 62% higher odds (crude OR = 1.616, 95% CI 1.098–2.380). Divorced participants had 53% lower crude odds than married participants (crude OR = 0.475, 95% CI 0.306–0.737, *p* < 0.001)—a protective association. Participants in the lowest income bracket had significantly higher crude odds than the highest earners (crude OR = 1.418, 95% CI 1.053–1.911).

**Table 4:** Binary logistic regression—crude and adjusted odds ratios for limited health literacy (*n* = 1,767).

Predictor	Crude OR (95% CI)	*p*	Adjusted OR (95% CI)	*p*	Interpretation
Self-rated health (ref: excellent)					
Poor	**14.618 (3.162–67.583)**	**<0.001**	**12.407 (2.616–58.840)**	**0.002**	Large but imprecise; small poor-SRH group
Fair	4.660 (2.870–7.564)	<0.001	4.400 (2.630–7.360)	<0.001	Graded association
Good	2.406 (1.823–3.176)	<0.001	2.468 (1.833–3.322)	<0.001	Graded association
Very good	1.742 (1.365–2.222)	<0.001	1.849 (1.433–2.387)	<0.001	Graded association
Age (per year)					
Continuous	0.995 (0.989–1.002)	0.172	**0.989 (0.982–0.997)**	**0.010**	Protective (OR 0.989/year)
Gender (ref: female)					
Male	1.111 (0.885–1.395)	0.366	1.113 (0.866–1.430)	0.404	NS
Nationality (ref: Kuwaiti)					
Non-Kuwaiti	1.149 (0.895–1.474)	0.276	1.001 (0.736–1.362)	0.995	NS
Bedoon	1.158 (0.584–2.295)	0.675	1.021 (0.496–2.102)	0.955	NS
Education (ref: Bachelor’s)					
Below secondary	1.813 (0.990–3.322)	—	0.930 (0.433–2.000)	0.853	NS
Secondary/diploma	1.167 (0.955–1.426)	—	1.164 (0.938–1.445)	0.168	NS
Postgraduate	0.928 (0.628–1.372)	—	1.089 (0.723–1.639)	0.684	NS
Income (ref: 2,000+ KD)					
<500 KD	**1.418 (1.053–1.911)**	0.034	1.316 (0.903–1.918)	0.154	NS after adjustment
500–999 KD	1.238 (0.904–1.694)	—	1.143 (0.796–1.640)	0.469	NS
1,000–1,499 KD	1.087 (0.842–1.404)	—	1.153 (0.874–1.522)	0.313	NS
Chronic disease (ref: no)					
Yes	1.229 (0.989–1.527)	0.062	1.122 (0.870–1.447)	0.374	NS
Internet use (ref: moderate)					
Low use	0.944 (0.711–1.253)	—	0.984 (0.734–1.319)	0.914	NS
High use	0.936 (0.758–1.156)	—	0.920 (0.740–1.143)	0.451	NS
Model fit			Fully adjusted		
Model *χ*^2^	—		82.327 (df = 18)	<0.001	*p* < 0.001
Nagelkerke *R*^2^	—		0.062		
Hosmer–Lemeshow	—		12.769 (df = 8)	0.120	Acceptable fit

aOR, adjusted odds ratio; CI, confidence interval; NS, not significant; ref, reference category. SRH showed the strongest and most consistent association across all models. Internet-use frequency was missing for 79 participants (4.3%); regression analyses were conducted among participants with complete covariate data (*n* = 1,767). Income data were complete for all participants. Bold = *p* < 0.05. H–L, Hosmer–Lemeshow test.

**Figure 1:**
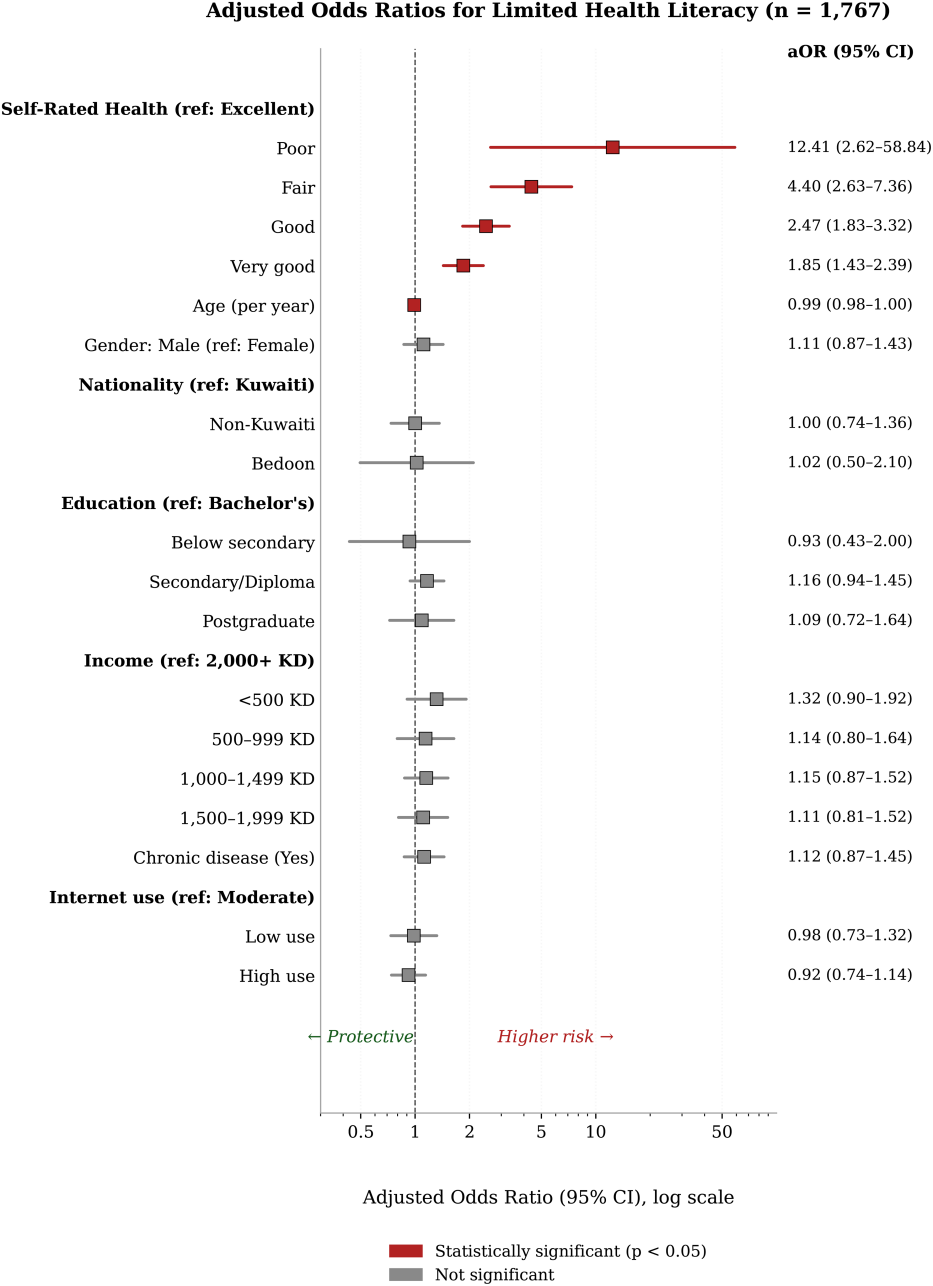
Adjusted odds ratios (95% CI) for limited health literacy from the fully adjusted binary logistic regression model (*n* = 1,767). Self-rated health categories show the steepest graded gradient. Squares represent point estimates; horizontal lines represent 95% confidence intervals on a log scale. The vertical dashed line at OR = 1 represents no association. Red markers indicate statistical significance (*p* < 0.05); grey markers indicate non-significant predictors.

The fully adjusted model was statistically significant and demonstrated good calibration (*χ*^2^ = 82.327, df = 18, *p* < 0.001; Nagelkerke *R*^2^ = 0.062; Hosmer–Lemeshow *p* = 0.120). Self-rated health was the factor most strongly associated with limited health literacy, retaining a clear graded association after full adjustment. Compared with participants reporting excellent health, the graded pattern persisted across all levels (fair: aOR = 4.400; good: aOR = 2.468; very good: aOR = 1.849; all *p* < 0.001). The point estimate for the poor category was large but imprecise, reflecting its small size (*n* = 12; aOR = 12.41, 95% CI 2.62–58.84, *p* = 0.002); sensitivity analyses described below confirmed the robustness of the overall gradient rather than this single estimate. Increasing age was independently associated with lower odds of limited HL (aOR = 0.989 per year, 95% CI 0.982–0.997, *p* = 0.010). All remaining factors—gender, nationality, education, income, chronic disease, and internet use—were non-significant after adjustment (all *p* > 0.374). The crude income association was fully attenuated after adjustment for health-related variables; because formal mediation was not tested, this attenuation is reported as an observation rather than evidence of a mediating pathway. Adjusted odds ratios for all predictors are visualised in Figure 1.

Sociodemographic variables alone failed to predict limited health literacy (Nagelkerke *R*^2^ = 0.011; *χ*^2^ = 14.303, df = 11, *p* = 0.217). Adding health-related variables produced a five-fold increase in explained variance (Δ*R*^2^ = 0.051; Δ*χ*^2^ = 68.024, *p* < 0.001), suggesting that health-related factors appeared to explain more variation in health literacy than sociodemographic characteristics in this sample.

Sensitivity analyses addressed the small poor-SRH category (*n* = 12). Collapsing the poor and fair categories yielded a stable adjusted odds ratio of 4.91 (95% CI 3.00–8.03, *p* < 0.001) for poorer (poor/fair) versus excellent self-rated health, with the graded pattern across the remaining categories unchanged (good: aOR = 2.47; very good: aOR = 1.85). Variance-robust and distribution-free tests of the bivariate association were also confirmatory (Welch *F* = 17.438, *p* < 0.001; Kruskal–Wallis *H* = 94.754, *p* < 0.001; Supplementary Table S1). Together, these analyses indicate that the graded association between poorer self-rated health and limited health literacy is robust, even though the magnitude of the isolated poor-category estimate should be interpreted with caution.

### 3.5 Subgroup and chronic disease analyses

Exploratory subgroup analyses showed broadly similar patterns; because some subgroups were small (e.g., Bedoon *n* = 35), these are interpreted with caution. Among Kuwaiti nationals (*n* = 1,497), the graded association between SRH and health literacy was replicated with comparable magnitude (*F* = 18.236, *p* < 0.001; poor: 8.60, excellent: 13.74). The age group effect was more pronounced within this subgroup (*F* = 6.438, *p* < 0.001), with adults aged 30–44 years scoring highest (13.47) and those aged 18–29 years lowest (12.54). Gender and education were non-significant within Kuwaiti nationals (*p* = 0.321 and 0.191, respectively). Among non-Kuwaiti residents (*n* = 314), the SRH gradient remained present (poor: 8.00, excellent: 13.51) and gender differences were negligible (male: 12.62 vs. female: 12.60). The Bedoon subgroup (*n* = 35) had a mean HLS score of 13.00 (SD = 3.46); inferential statistics are not reported for this group given the small sample size.

Within the 453 participants with chronic disease, some variation by chronic disease subtype was observed. Obesity showed the largest observed HL deficit, although the effect size was small: participants with obesity scored 1.22 points lower than those without (11.76 vs. 12.98; *F* = 14.496, *p* < 0.001, *η*^2^ = 0.008). Hypertension (*F* = 5.910, *p* = 0.015) and diabetes (*F* = 4.464, *p* = 0.035) were also associated with lower scores, while heart disease (*p* = 0.157) and asthma (*p* = 0.553) were not statistically significant. These findings suggest that HL interventions may be particularly warranted for adults living with obesity and metabolic conditions, where the gap between information need and HL capacity is greatest.

## 4 Discussion

This study provides one of the first large-scale, venue-based assessments of health literacy among adults in Kuwait, using the internationally validated HLS-EU-Q16 in a sample of 1,846 adults surveyed across all six governorates. The principal findings are fourfold: more than one in three surveyed adults (37.2%) had limited health literacy; finding information on mental illness was the most difficult task by a clear margin; self-rated health was the factor most strongly associated with limited health literacy, showing a graded relationship that persisted after full multivariable adjustment and was robust to assumption-free sensitivity analyses; and sociodemographic variables—including gender, nationality, education, and income—were not independently associated with health literacy once health-related factors were included. Because recruitment was venue-based and the achieved sample was predominantly Kuwaiti and female, these findings characterise the surveyed population rather than all adults residing in Kuwait.

The proportion of surveyed adults with limited health literacy (37.2%) is broadly consistent with estimates from European populations, where the original HLS-EU survey reported approximately 47% of adults having limited health literacy across eight countries (19), and with findings from Middle Eastern and Central Asian settings (20). This figure is, however, higher than would be expected for a high-income country with universal primary healthcare access, suggesting that healthcare provision alone may be insufficient to ensure adequate health literacy among surveyed adults. The inadequate category (10.6%) is comparable to rates reported in other GCC populations (9). These findings reinforce the recognition that limited health literacy remains a persistent public health challenge across diverse socioeconomic settings (9).

The identification of self-rated health as the factor most strongly associated with limited health literacy in the adjusted model is a key finding of this study. The graded gradient from poor SRH (mean HLS 8.50) to excellent SRH (mean 13.70) spans one-third of the scale range. Because only 12 participants reported poor SRH, the large adjusted odds ratio for that single category was imprecise (95% CI 2.62–58.84); a sensitivity model collapsing the poor and fair categories produced a more stable estimate (aOR 4.91, 95% CI 3.00–8.03). The robustness of the overall graded pattern, rather than the magnitude of any single category, is therefore the dependable finding. This pattern is consistent with prior evidence that individuals with lower health literacy are more likely to report poorer health status (4, 30). Several mechanisms may underpin the relationship: individuals with limited health literacy may have reduced capacity to interpret medical information, adhere to treatment plans, and engage in preventive behaviours, contributing to poorer perceived health (5, 30); conversely, individuals with poorer health encounter greater informational complexity in healthcare interactions (31). Importantly, both SRH and health literacy were self-reported and measured at the same time point, so common-method bias, reverse causation, and residual confounding cannot be excluded, and the cross-sectional design precludes any causal or directional inference (5). Whether SRH could serve as a practical screening indicator for limited health literacy is a hypothesis for future research; establishing this would require prospective designs and formal assessment of discrimination (e.g., sensitivity, specificity, or area under the curve), which were beyond the scope of this study.

Socioeconomic factors, particularly income, showed significant crude associations with limited health literacy, with participants in the lowest income bracket carrying 42% higher unadjusted odds than the highest earners. This is consistent with extensive evidence linking socioeconomic disadvantage to reduced health literacy (6). Financial constraints may restrict access to health information resources, digital tools, and educational opportunities that support health literacy development (6). In the Kuwaiti context, income disparities may also reflect differential access to private healthcare and diverse information channels. The income effect was, however, fully attenuated after adjustment for health-related variables. This attenuation suggests that health-related factors may partly account for the observed relationship; however, formal mediation was not tested and this interpretation should be treated as exploratory.

The association between chronic disease and lower health literacy at the bivariate level is consistent with prior research demonstrating that individuals with chronic conditions face heightened informational and self-management demands (31). Limited health literacy may impair patients’ ability to interpret complex treatment instructions, engage effectively with providers, and navigate healthcare systems, contributing to suboptimal disease management outcomes (31). The finding that obesity was the single chronic disease subtype most strongly associated with lower health literacy (*F* = 14.496, *p* < 0.001, *η*^2^ = 0.008) extends the existing literature and highlights unique challenges for this group in accessing and applying health information about nutrition, physical activity, and metabolic risk.

The mental health information gap identified in this study—with only 57% of participants finding C8 (information on depression and stress) easy, 13 percentage points below the next hardest item—is both clinically important and actionable. Mental health literacy—defined by Jorm et al. (32) as the knowledge and beliefs about mental disorders that aid recognition, management or prevention—is increasingly recognised as distinct from general health literacy and is particularly limited in settings where mental health stigma constrains open discourse (7). Kuwait, like many GCC countries, has historically had limited public engagement with mental health as a health topic, and specialist mental health services are under-resourced relative to population need (8). C8 ease ratings were consistently low across nationality groups (Kuwaiti 56.2%, non-Kuwaiti 58.6%, Bedoon 54.3% rating it easy), sexes (males 60.4%, females 55.6%), and age groups (range 51.6–59.8%), with subgroup figures provided in Supplementary Table S2, supporting the sample-wide relevance of the mental health information gap. This consistency points to a sample-wide deficit in mental health information access that warrants attention in health communication strategies.

Education level was not independently associated with health literacy after adjustment. Although a positive trend was observed across educational levels in bivariate analysis, it was attenuated in the multivariable model. This pattern has been reported in prior studies and reflects the multidimensional nature of health literacy, which extends beyond educational attainment to include the specific capacity to critically evaluate and apply health information (7). In Kuwait, where educational attainment is relatively high but health literacy competencies vary substantially, formal education alone is insufficient as a proxy for health literacy.

Similarly, internet use for health information was not independently associated with health literacy after adjustment. While digital access is often assumed to facilitate health information acquisition, existing evidence indicates that access alone does not ensure effective or critical use of such information (33). This distinction between access and digital health literacy—the ability to critically evaluate online health content—is particularly pertinent in Kuwait, where high digital penetration coexists with limited health literacy in a substantial proportion of the population (8).

Age-group differences in health literacy were small and less consistent across sensitivity analyses: although younger adults (18–29 years) had somewhat lower HLS scores than those aged 30–44 years at the bivariate level, this difference was significant under Welch’s ANOVA (*p* = 0.010) but not under the distribution-free Kruskal–Wallis test (*p* = 0.062), indicating a weak and assumption-dependent association. The more reliable finding was the modest independent effect of continuous age in the adjusted model. To the extent that younger adults show lower health literacy despite presumed greater digital engagement, this would diverge from assumptions about digitally connected cohorts and is consistent with some prior evidence (34); students, for example, may have less practical experience with health decision-making than employed adults. These observations support integrating health literacy education into tertiary curricula and workplace health promotion programmes, while recognising that the age-group signal in these data was weak.

### 4.1 Implications for inclusive community health education and equity

These findings carry direct implications for community health education and health equity in Kuwait and the wider Gulf region. The persistence of limited health literacy in over one-third of this surveyed sample, despite universal healthcare access in Kuwait, suggests that service provision alone does not generate health literacy; community-level engagement and culturally responsive communication are required. Three priorities emerge from these data. First, community health education programmes should target individuals with poorer self-rated health and chronic conditions—particularly obesity and other cardiometabolic conditions (13, 14)—rather than relying on education or income as targeting criteria, since these were not independent predictors after adjustment. Second, the substantial gap in mental health information access points to an urgent need for stigma-sensitive, culturally appropriate, and linguistically accessible mental health communication, integrated into primary care and community settings. Third, the fact that internet use was not associated with higher health literacy after adjustment underscores that simply increasing digital access is insufficient; critical digital and media literacy skills are also required (33). Community-centred health education should therefore address digital, media, and navigation literacy as components of broader health equity, with attention to the diverse language needs of Kuwaiti, expatriate, and Bedoon communities. System-level responsiveness—the capacity of health organisations to make services easier to access, understand, and navigate—will be essential to translate these findings into equitable health outcomes. Organisational health literacy frameworks emphasise that responsibility for health literacy rests not only with individuals but also with the systems that serve them (35, 36); practical measures include plain-language patient information, multilingual health communication, and routine attention to health literacy in primary care.

### 4.2 Strengths and limitations

This study has several methodological strengths. The sample size of 1,846 participants is among the largest health literacy surveys conducted in the Gulf region, providing robust statistical power for adjusted analyses and subgroup exploration across nationality strata. Recruitment spanned all six governorates of Kuwait, enhancing geographic and demographic diversity. The use of both validated Arabic and English versions of the HLS-EU-Q16, with parallel data cleaning protocols, supports inclusion of Arabic- and English-speaking residents and enables cross-cultural comparison with European and Asian benchmarks. Internal consistency was excellent (Cronbach’s *α* = 0.849), and analyses included crude and adjusted regression models with reporting of effect sizes (*η*^2^, partial *η*^2^, Nagelkerke *R*^2^), model fit statistics, and hierarchical block-entry models, in line with current STROBE reporting standards (24).

Several limitations should be acknowledged. First, and most importantly, the sample is not representative of the Kuwaiti resident population. Recruitment used quota sampling by governorate, nationality, and age group combined with convenience recruitment at public venues; the achieved sample was 81.1% Kuwaiti and 78.4% female, whereas national statistics indicate that Kuwaiti nationals constitute approximately 31% of residents. The questionnaire was available only in Arabic and English, which under-represents the substantial expatriate population not literate in either language. The prevalence estimate and all associations therefore apply to the Arabic- and English-literate, venue-based sample surveyed, not to all adults residing in Kuwait, and the title, abstract, and conclusions have been framed accordingly. Quota targets were set but were not uniformly achieved, and the total number of individuals approached and the number who declined were not systematically recorded, which prevents calculation of a response rate; this is acknowledged as a limitation. A participant flow diagram is provided in Supplementary Figure S1. Second, the cross-sectional design precludes causal inference, and the observed SRH–health literacy relationship may be bidirectional. Both the primary outcome and the strongest associated factor (SRH) were measured by self-report at a single time point, so common-method bias may have inflated the strength of this association. Third, the female majority (78.4%) is consistent with female over-representation commonly documented in Gulf health surveys (9) but limits generalisability to men; sex-stratified analyses were not performed. Fourth, diagnostic checks indicated heterogeneity of variance (Levene’s *p* < 0.001) and non-normal residuals for the bounded HLS-EU-Q16 score; although the large sample size renders the parametric *F*-tests reasonably robust, key bivariate comparisons were therefore re-examined using Welch’s ANOVA, Games–Howell *post-hoc* tests, and Kruskal–Wallis or Mann–Whitney tests, which confirmed the main findings. Fifth, the adjusted models explained a modest proportion of variance (Nagelkerke *R*^2^ = 0.062), indicating that important unmeasured psychosocial, communication, and system-level factors remain; future studies should incorporate health self-efficacy, patient activation, and prior health education experience. Finally, internet-use frequency data were missing for 4.3% of participants and were handled through complete-case analysis in models including this covariate; income data were complete for all participants.

That sociodemographic variables were not independently associated with limited health literacy in the multivariable model—despite individually significant bivariate associations—may suggest that their apparent effects operate partly through health-related pathways, although this was not formally tested and the overall model explained only a modest proportion of variance. This observation, interpreted cautiously, has potential implications for intervention design: targeting health literacy programmes on the basis of education level or income alone may be less efficient than approaches that also consider health status indicators, chronic disease diagnoses, and self-reported health confidence. This is particularly relevant in Kuwait, where national calls for action on NCD prevention and health promotion have emphasised the need for targeted, evidence-based interventions (14).

## 5 Conclusion

Over one-third of the adults surveyed in Kuwait had limited health literacy, despite the country’s high-income status and universal healthcare provision. Self-rated health was the factor most strongly associated with limited health literacy in the adjusted model, showing a graded relationship that persisted after full multivariable adjustment and was robust to assumption-free sensitivity analyses; the cross-sectional design, however, precludes causal or directional inference. Mental health information access represents a specific, actionable, and cross-cutting gap that warrants dedicated community-based health communication. These findings call for an integrated response that combines accessible community health education, culturally and linguistically appropriate communication, system-level responsiveness, and targeted support for individuals with chronic conditions and poorer perceived health (13, 14). Because the sample was venue-recruited and predominantly Kuwaiti and female, these conclusions apply to the surveyed population rather than all residents of Kuwait; future research should employ probability-based, multilingual sampling, investigate longitudinal pathways between health status and health literacy, and evaluate community-based and digital health literacy interventions. With these caveats, the findings may help inform national health literacy strategies, multilingual public health communication frameworks, and equity-oriented NCD prevention initiatives in Kuwait and the broader Gulf region.

## Data Availability

The datasets presented in this article are not readily available because the datasets generated and analysed during the current study are not publicly available due to restrictions specified in the ethical approval from the Kuwait Ministry of Health (Research #3079/2025), but anonymised data may be made available from the corresponding author (ahmad.salman@ku.edu.kw) upon reasonable request and subject to appropriate institutional and ethical clearance. Requests to access the datasets should be directed to ahmad.salman@ku.edu.kw.
