# Supplementary material for: Health literacy and its correlates among adults surveyed in Kuwait: a venue-based cross-sectional study
Source: Front Public Health. 2026 Jul 8;14:1878619. doi: 10.3389/fpubh.2026.1878619 (PMC13388251; doi:10.3389/fpubh.2026.1878619)
Supplement: Supplementary file 1 [file Data_Sheet_1.PDF]

Supplementary Material

Health Literacy and Its Correlates Among Adults Surveyed in Kuwait: A Venue-Based Cross-Sectional Study

Supplementary Figure S1. Participant flow

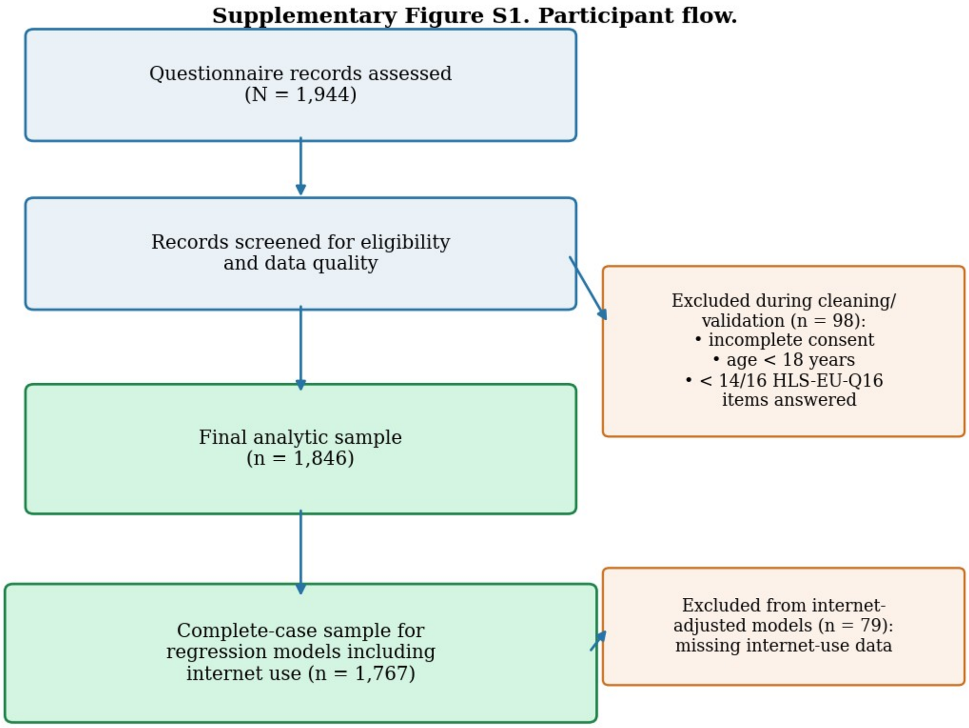

Supplementary Figure S1. Flow of participants from initial questionnaire records (N = 1,944) to the final analytic sample (n = 1,846) and the complete-case sample for internet-adjusted regression models (n = 1,767). Because the questionnaire was distributed electronically, the number of individuals who viewed but did not complete it could not be determined; detailed numeric recruitment targets and refusal counts were not systematically retained.

Supplementary Table S1. Robustness (variance-robust and distribution-free) sensitivity analyses for key bivariate associations with HLS-EU-Q16 score

Predictor	One-way ANOVA	Welch ANOVA	Kruskal–Wallis	Interpretation
**Self-rated health**	F = 22.388, p < .001	F = 17.438 (df 4, 76.4), p < .001	H = 94.754, df 4, p < .001	Robust; graded gradient confirmed
**Marital status**	F = 4.738, p = .003	F = 5.433 (df 3, 163.4), p = .001	H = 14.616, df 3, p = .002	Robust
**Employment status**	F = 3.318, p = .005	F = 3.228 (df 5, 251.2), p = .008	H = 20.206, df 5, p = .001	Robust
**Age group**	F = 3.623, p = .013	F = 3.797 (df 3, 607.4), p = .010	H = 7.316, df 3, p = .062	Weak; not significant under Kruskal–Wallis
**Chronic disease (binary)**	t = 2.711, p = .007	Welch t = 2.619 (df 725.9), p = .009	Mann–Whitney U, Z = −2.819, p = .005	Robust

Note. Welch ANOVA does not assume equal variances; Kruskal–Wallis and Mann–Whitney tests are rank-based distribution-free sensitivity analyses. These were conducted because Levene's test indicated heterogeneity of variance (p < .001) and residuals were non-normal (Shapiro–Wilk p < .001) for the bounded HLS-EU-Q16 score. The self-rated health gradient and the marital, employment, and chronic-disease associations were robust across all tests; the age-group association was significant under parametric and Welch tests but not under the Kruskal–Wallis test.

Supplementary Table S2. Item C8 (finding information on mental illness): percentage rating the task easy/very easy, by subgroup

Subgroup	n	% rating C8 easy/very easy
**Overall**	1,846	56.6%
**Nationality: Kuwaiti**	1,497	56.2%
**Nationality: Non-Kuwaiti**	314	58.6%
**Nationality: Bedoon**	35	54.3%
**Sex: Male**	399	60.4%
**Sex: Female**	1,447	55.6%
**Age 18–29**	652	59.8%
**Age 30–44**	571	57.3%
**Age 45–59**	469	51.6%
**Age 60+**	154	55.8%

Note. C8 ease ratings were consistently low across nationality, sex, and age subgroups (range 51.6–60.4%), supporting the sample-wide relevance of the mental health information gap. For reference, the next most difficult item overall (C11, judging media information) was rated easy by 69.6% of the full sample.
